# Supplementary material for: MaAts, an Alkylsulfatase, Contributes to Fungal Tolerances against UV-B Irradiation and Heat-Shock in Metarhizium acridum
Source: J Fungi (Basel). 2022 Mar 8;8(3):270. doi: 10.3390/jof8030270 (PMC8951457; doi:10.3390/jof8030270)
Supplement: Supplementary file 1 [file jof-08-00270-s001.zip › jof-1614201-supplementary.pdf]

MaAts, an Alkylsulfatase, Contributes to Fungal Tolerances against UV-B Irradiation and Heat-Shock in *Metarhizium acridum*

Lei Song [1,2,3,†], Xiaoning Xue [1,2,3,†], Shuqin Wang [1,2,3], Juan Li [1,2,3], Kai Jin [1,2,3,*] and Yuxian Xia [1,2,3,*]

1 Genetic Engineering Research Center, School of Life Sciences, Chongqing University, Chongqing 401331, China; 201826021018@cqu.edu.cn (L.S.); 201726021003@cqu.edu.cn (X.X.); 202026021000@cqu.edu.cn (S.W.); 201626022016@cqu.edu.cn (J.L.)
2 Chongqing Engineering Research Center for Fungal Insecticide, Chongqing 401331, China
3 Key Laboratory of Gene Function and Regulation Technologies under Chongqing Municipal Education Commission, Chongqing 401331, China
* Correspondence: jinkai@cqu.edu.cn (K.J.); yuxianxia@cqu.edu.cn (Y.X.); Tel.: +86-23-65120990 (Y.X.)
† These authors contributed equally to this work.

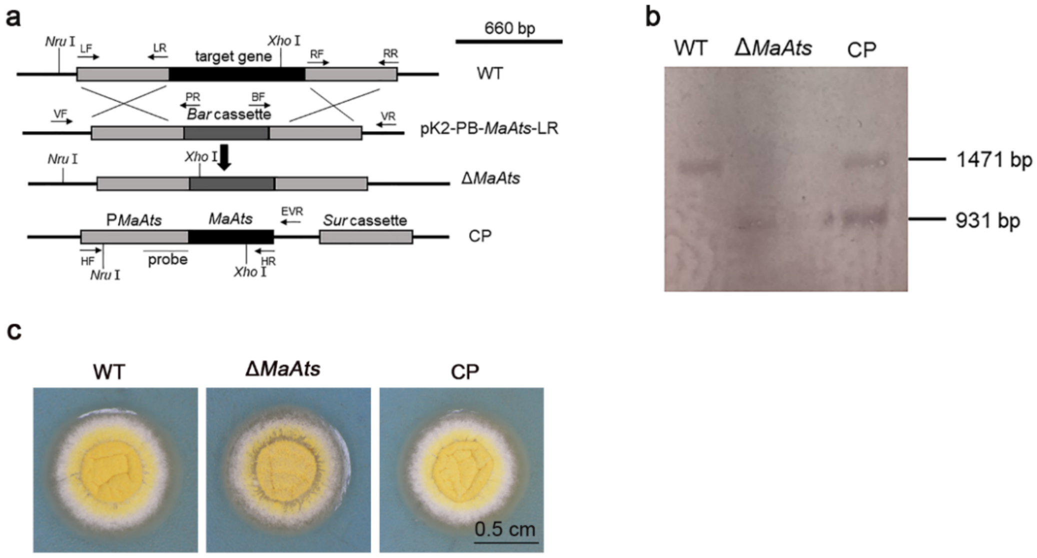

Figure S1 Deletion and complementation of *MaAts* in *M. acridum.* (a) Strategies of the *MaAts* deletion and complementation in *M. acridum*. (b) Southern blotting. The genomic DNA from different fungal strains was cut with *Nru* I and *Xho* I. The probe was cloned from 5′ genomic sequence of *MaAts* by PCR using the primers of MaAts-PF/MaAts-PR (Table S1). (c) Colonies of each strain on 1/4 SDAY plates at 28°C for 6 days.

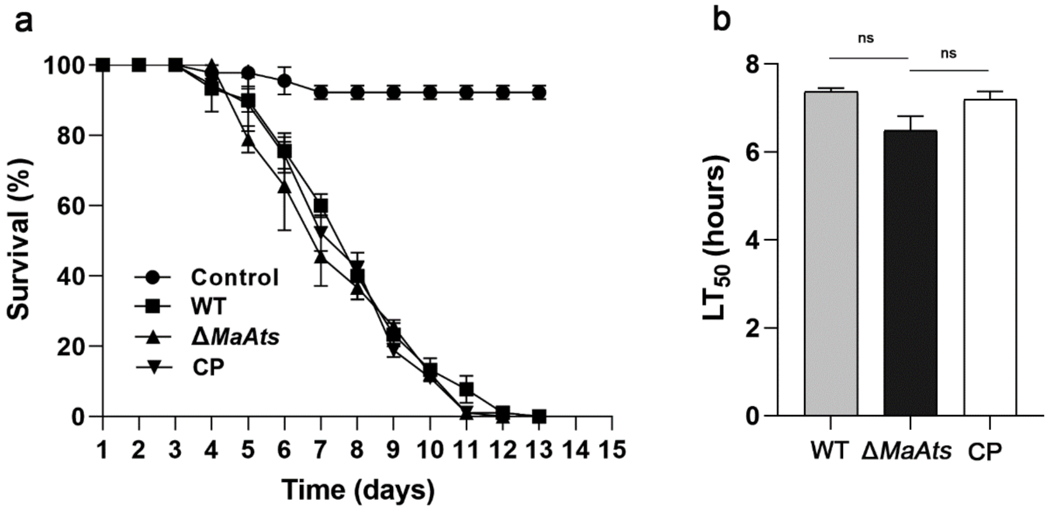

Figure S2 Bioassays. (a) Survival of locusts after topical inoculation. Locusts treated by paraffin oil was used as control. (b) The mean 50% lethal time (LT_{50}) of fungal strains. ns indicates P > 0.05.

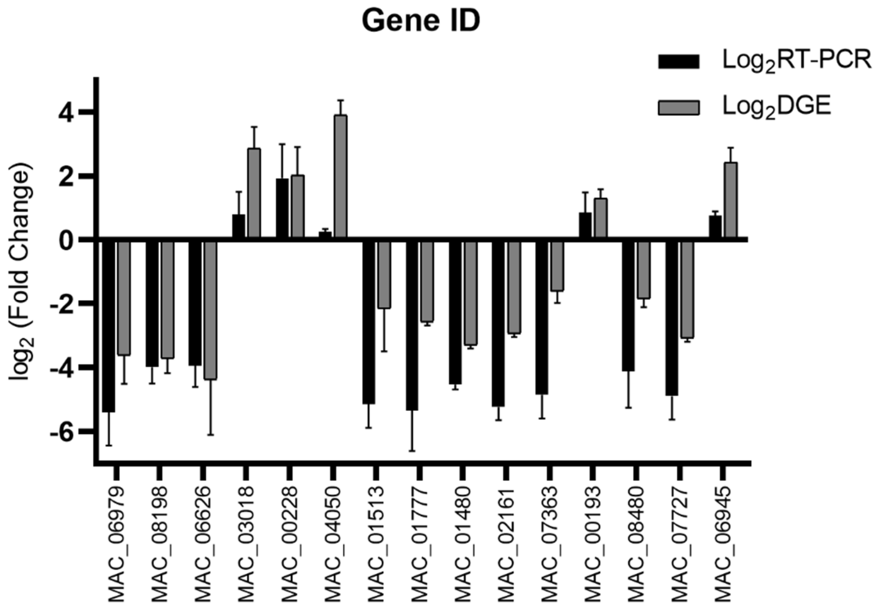

Figure S3 Verification of the DGE data via qRT-PCR. All the primers for qRT-PCR were presented in Table S1.

Table S1 Primers used in this study.

Primers	Sequence (5′-3′)*	Restriction enzyme sites	Remarks
MaAts-LF	GACGGCCAGTGCCAAGCTTCTTCTTGCTGCTTTCGC	*Hin*d III	To clone the 5′ and 3′ flanking sequences of the *MaAts* gene for the disruption of *MaAts*
MaAts-LR	CGGATCCCTCGAGTCTAGAGCAAAGCGGTATCCATC	*Xba* I	
MaAts-RF	ACCGAGATCTAATAAGATACAACTCCGTGCCTTCATCC	*Eco*R V	
MaAts-RR	ATGACATGATTACGAATTGACTGGGTAGCAGCGGTAAG	*Eco*R I	
Bar-F	GCTCTACACCCACCTGCT		To screen the Δ*MaAts* strain
MaAts-VR	CGACGAGATTGAAAGCCTACAT		
Pt-R	CAGCCAAGCCCAAAAAGTG		To screen the Δ*MaAts* strain
MaAts-VF	GCTGGTAAACGCCGTCAAT		
MaAts-HF	GACGGCCAGTGCCAAGCTTTCTTCTTGCTGCTTTCGC	*Hin*d III	To clone *MaAts* sequence for complementation
MaAts-HR	CCTTGCTCACCATGGATCCCGGCGTAACAATATCAAAGTTCC	*Bam*H I	
MaAts-CP-VF	GCCATCGTCTACACGCACTG		To screen the *MaAts* complementation strain
EGFP-VR	CGATGCGGTTCACCAGGGTGT		
MaAts-PF	CACTTCAGTCCGTGTTCCC		Clone the probe
MaAts-PR	TGTTTGATTCCATGCTGCTAG		
MAC_06979-QF	CGCAGGCACCGATAGTAACGATG		Used to validate the data of DGE by qRT-PCR
MAC_06979-QR	TTAACACGACGGTTCAGCCACAC		
MAC_08198-QF	GACCACTCCAGACAACCAGCAAC		
MAC_08198-QR	CAGCGGAGCAGATGGAATGTTAGG		
MAC_06626-QF	GAACCATACCATTCAGACCCCATCC		
MAC_06626-QR	CGGCCCTTTGTATCATACCAGTAGC		
MAC_03018-QF	GCCAGGTTGTCCAATCCAGAAGTC		
MAC_03018-QR	CGAGAGCCAAGAGAAATGAAATGCG		
MAC_00228-QF	AGGCGAGATGATTTCTGGCTCAAAG		
MAC_00228-QR	CAGGCGAGTGTCAGCGATGATAG		
MAC_04050-QF	GGTAGGTGGAATCGGGCTAACAATG		
MAC_04050-QR	TGCGGAGATTTGGTTCATCAGGAAG		
MAC_01513-QF	TTCTGCACCTGCGATGTAATCTCAC		
MAC_01513-QR	TGAATGGCGGCGAAGATAGAAGC		
MAC_01777-QF	TCCTATCCCTGGTTGCCCTGATC		
MAC_01777-QR	GCTGCCGCTCTCGACATATTGG		
MAC_01480-QF	GCTGCCATTGACGAGTATGAGGAG		
MAC_01480-QR	GTCTCTTCAACACTGCCGACTTCTC		
MAC_02161-QF	GTGGGACAGGACCAAGAAGGTAAAC		
MAC_02161-QR	TTTGCCTCGTGGGTTCGTAGTTG		
MAC_07363-QF	ATCATGTGCGACGGCGTTGTA		
MAC_07363-QR	CACGAGTTCCTCCAAAGTCCATACC		
MAC_00193-QF	GATTGACGAGGAGAGTGGTGAAGTG		
MAC_00193-QR	AAGGGAGACGGTTACGAGGATGAG		

MAC_08480-QF	AGTTTACCAACCAGAGCCGCAAG
MAC_08480-QR	TTCCCTTCCTCCGTGAACCAGTC
MAC_07727-QF	GGACGACACAAGGACGACTATCAAC
MAC_07727-QR	ATTAGCACTCAGCCCCAACGAAAG
MAC_06945-QF	ACCAACACGATATTTGCTGCCCTAC
MAC_06945-QR	TGTCGGAGTTGTTAGCAGTGATGTC
Gapdh-QF	AGATGGAGGAGTTGGTGTTG
Gapdh-QR	GACTGCCCGCATTGAGAAG

*Underlined sequences are restriction sites.

Table S2 DGE data of Δ*MaAts* vs. WT.

Gene ID	Log_2^{ratio} (Δ*MaAts*/WT)	*q* value	Up or down regulation	Description
MAC_08440	-9.30485	2.42E-13	down	SPX domain protein
MAC_02161	-4.25453	0.008879	down	hypothetical protein
MAC_06623	-3.92455	1.4E-113	down	hypothetical protein
MAC_02079	-3.77232	0.035573	down	integral membrane protein
MAC_06626	-3.72527	1.21E-11	down	hypothetical protein
MAC_08198	-3.5826	1.28E-32	down	extracellular dioxygenase, putative
MAC_08889	-3.4474	8.98E-47	down	nucleoside-diphosphate-sugar epimerase family protein
MAC_06979	-3.44465	1.69E-07	down	C6 transcription factor, putative
MAC_08891	-3.41733	1.52E-38	down	Maleylacetate reductase, putative
MAC_06990	-3.35268	7.77E-06	down	putative glycosyltransferase family 2 protein
MAC_09509	-3.3455	2.80E-04	down	hypothetical protein
MAC_06293	-3.28064	4.20E-32	down	beta-lactamase family protein
MAC_09765	-3.19806	2.70E-06	down	phosphatidylserine decarboxylase family protein
MAC_01480	-3.14834	1.06E-68	down	phosphoinositide 3-phosphate phosphatase
MAC_00187	-3.11194	0.019044	down	hypothetical protein
MAC_01452	-3.09371	7.09E-24	down	phospholipase A-2-activating protein
MAC_05898	-3.05505	2.45E-14	down	MFS transporter, putative
MAC_02138	-2.93381	2.01E-22	down	Heat Labile Enterotoxin Type Iib
MAC_07727	-2.92254	6.00E-64	down	hypothetical protein
MAC_02602	-2.88954	1.04E-06	down	protein tyrosine phosphatase
MAC_00186	-2.87686	1.59E-15	down	aprataxin-like protein
MAC_05216	-2.85544	3.8E-116	down	WSC domain containing protein
MAC_09732	-2.72105	0.043849	down	hypothetical protein
MAC_04005	-2.65364	7.13E-41	down	putative maltase MLT2
MAC_04973	-2.62274	4.63E-40	down	cytochrome P450, putative
MAC_04112	-2.55617	2.60E-20	down	cysteine synthase K/M:Cysteine synthase B
MAC_07559	-2.5519	2.78E-04	down	beta-glucosidase
MAC_04117	-2.55183	2.54E-48	down	hypothetical protein
MAC_02353	-2.51247	1.86E-30	down	hypothetical protein
MAC_06307	-2.44318	5.67E-29	down	rab GDP-dissociation inhibitor
MAC_08505	-2.41638	7.23E-15	down	C2H2 transcription factor
MAC_08201	-2.41358	2.10E-08	down	hypothetical protein
MAC_09507	-2.41069	8.05E-11	down	ankyrin repeat domain containing protein
MAC_05587	-2.4044	1.07E-41	down	hypothetical protein

MAC_02763	-2.38707	1.91E-27	down	tetraspanin
MAC_01777	-2.38397	8.62E-22	down	MYND finger family protein
MAC_05376	-2.37816	6.73E-05	down	C2H2 type zinc finger domain containing protein
MAC_08200	-2.33103	2.68E-34	down	hypothetical protein
MAC_02951	-2.2094	6.56E-49	down	succinate-semialdehyde dehydrogenase, putative
MAC_08447	-2.19101	1.83E-36	down	krev-1
MAC_06988	-2.18956	1.40E-06	down	DNA-binding protein SMUBP-2
MAC_01216	-2.18362	0.004813	down	DUF341 domain protein
MAC_04111	-2.16213	1.70E-25	down	hypothetical protein
MAC_06987	-2.11299	4.58E-06	down	cystathionine gamma-synthase, putative
MAC_06983	-2.07005	8.04E-10	down	cytochrome P450, putative
MAC_06981	-2.05974	0.001023	down	phytanoyl-CoA dioxygenase family protein
MAC_05633	-2.05375	1.14E-06	down	MedA
MAC_02952	-2.04546	4.14E-21	down	dihydroxyacetone kinase
MAC_02757	-2.04089	4.83E-52	down	hypothetical protein
MAC_02625	-2.0284	1.60E-14	down	hypothetical protein
MAC_01254	-2.02161	5.14E-46	down	hypothetical protein
MAC_08196	-2.01644	1.16E-23	down	cytochrome P450, putative
MAC_07565	-2.01167	1.01E-20	down	oxidoreductase, 2OG-Fe(II) oxygenase family, putative
MAC_01513	-2.01018	4.17E-04	down	polyketide synthase, putative
MAC_09269	-1.99958	0.039197	down	DUF341 domain protein
MAC_02137	-1.9948	7.81E-04	down	integral membrane protein
MAC_04072	-1.97564	7.82E-07	down	lipoxygenase
MAC_06625	-1.94679	4.54E-22	down	hypothetical protein
MAC_06989	-1.942	1.31E-13	down	MFS transporter
MAC_06296	-1.9258	5.22E-10	down	metallopeptidase MepB
MAC_07769	-1.90303	1.33E-12	down	Mmc protein
MAC_08199	-1.89429	1.04E-17	down	hypothetical protein
MAC_01109	-1.89184	5.28E-40	down	delta-9 fatty acid desaturase
MAC_09694	-1.88945	1.33E-28	down	D-amino-acid oxidase
MAC_04508	-1.88312	0.003451	down	NlpC/P60-like cell-wall peptidase
MAC_05677	-1.88308	2.88E-21	down	Pfs, NACHT and Ankyrin domain protein
MAC_06776	-1.85887	2.56E-14	down	hypothetical protein
MAC_06982	-1.83408	4.09E-07	down	hypothetical protein
MAC_04116	-1.82859	7.68E-52	down	monooxygenase, putative
MAC_06274	-1.81219	1.12E-08	down	ABC multidrug transporter, putative
MAC_07130	-1.80302	1.69E-13	down	RTA1 domain protein, putative
MAC_05432	-1.77886	0.00913	down	monooxygenase, putative

MAC_09436	-1.75944	1.29E-28	down	hypothetical protein
MAC_06000	-1.75076	3.27E-35	down	MFS monosaccharide transporter, putative
MAC_05574	-1.73463	9.01E-05	down	C4-dicarboxylate transporter, putative
MAC_09776	-1.7303	0.004898	down	ATP-dependent RNA helicase dbp9
MAC_07499	-1.72499	5.68E-07	down	hypothetical protein
MAC_08480	-1.72229	1.13E-23	down	white collar 1
MAC_01512	-1.71032	1.27E-07	down	hypothetical protein
MAC_04635	-1.70656	9.20E-07	down	hypothetical protein
MAC_08474	-1.70206	2.30E-17	down	hypothetical protein
MAC_06821	-1.69528	3.58E-04	down	guanyl-specific ribonuclease F1
MAC_04393	-1.69149	1.13E-05	down	exo-beta-1,3-glucanase
MAC_00227	-1.68301	0.013158	down	ankyrin repeat-containing protein, putative
MAC_02349	-1.67268	1.01E-36	down	Guanyl-specific ribonuclease F1
MAC_06985	-1.66877	1.95E-11	down	meiotically up-regulated protein
MAC_05502	-1.66648	2.12E-06	down	elongator complex protein
MAC_04267	-1.66561	3.72E-26	down	hypothetical protein
MAC_05846	-1.66192	4.90E-06	down	WD repeat protein
MAC_01213	-1.65983	0.004645	down	indole-diterpene biosynthesis protein PaxU, putative
MAC_02351	-1.64931	1.85E-25	down	aldose-1-epimerase, putative
MAC_01091	-1.63546	6.84E-07	down	glucose transporter
MAC_02934	-1.63407	6.56E-05	down	MFS multidrug transporter, putative
MAC_01276	-1.62801	2.79E-15	down	quinone oxidoreductase
MAC_06986	-1.61156	1.07E-11	down	hypothetical protein
MAC_06980	-1.60712	1.20E-11	down	putative epoxide hydrolase
MAC_06984	-1.60247	6.28E-14	down	hypothetical protein
MAC_05835	-1.60089	1.65E-08	down	hypothetical protein
MAC_02666	-1.59778	1.99E-25	down	homogentisate 1,2-dioxygenase
MAC_07196	-1.59347	1.60E-43	down	hypothetical protein
MAC_05851	-1.59243	7.00E-11	down	salicylate hydroxylase
MAC_01217	-1.58953	2.36E-12	down	hypothetical protein
MAC_02485	-1.58177	5.02E-16	down	GMP synthase
MAC_00261	-1.57438	1.09E-10	down	hypothetical protein
MAC_04471	-1.56717	3.79E-06	down	ankyrin 2,3/unc44
MAC_07785	-1.56716	5.81E-20	down	hypothetical protein
MAC_02819	-1.56254	6.02E-08	down	Serine/threonine protein phosphatase 2A
MAC_09338	-1.55984	5.01E-15	down	cAMP-dependent protein kinase regulatory subunit
MAC_07472	-1.55087	3.66E-18	down	aldehyde dehydrogenase
MAC_03240	-1.54805	0.018293	down	peroxisomal copper amine oxidase
MAC_07641	-1.54608	2.75E-05	down	sodium/phosphate symporter, putative

MAC_03530	-1.54491	7.32E-18	down	glycerophosphoryl diester phosphodiesterase family protein
MAC_00031	-1.54157	3.34E-32	down	MFS multidrug transporter, putative
MAC_05894	-1.5344	6.98E-10	down	protein kinase-like protein
MAC_05138	-1.52627	9.07E-15	down	hypothetical protein
MAC_05814	-1.52363	8.11E-11	down	ATP-binding cassette transporter, putative
MAC_05916	-1.51631	6.41E-27	down	N,O-diacetyl muramidase, putative
MAC_08860	-1.51049	2.26E-30	down	hypothetical protein
MAC_05094	-1.50782	9.54E-04	down	hypothetical protein
MAC_09552	-1.50637	5.25E-05	down	hypothetical protein
MAC_00154	-1.50583	0.016488	down	hypothetical protein
MAC_07363	-1.50259	1.67E-08	down	hypothetical protein
MAC_04482	-1.49892	3.24E-04	down	acid phosphatase AphA
MAC_04115	-1.49733	1.24E-12	down	glycerol kinase, putative
MAC_00691	-1.49139	3.95E-09	down	C6 transcription factor
MAC_01215	-1.48518	8.83E-09	down	endonuclease/exonuclease/phosphatase family protein
MAC_03594	-1.48451	0.037723	down	C6 transcription factor, putative
MAC_03593	-1.48222	2.42E-04	down	siderophore biosynthesis enzyme, putative
MAC_05700	-1.48024	0.042427	down	serine-type carboxypeptidase
MAC_02808	-1.45801	1.30E-09	down	hypothetical protein
MAC_08803	-1.45686	3.55E-08	down	hypothetical protein
MAC_07549	-1.45633	0.004472	down	60S ribosome biogenesis protein Mak11, putative
MAC_05604	-1.45559	8.70E-14	down	2,3-dihydroxybenzoic acid decarboxylase dhbD
MAC_01197	-1.4538	1.54E-29	down	ADP-ribosylation factor
MAC_08795	-1.44625	2.36E-04	down	hypothetical protein
MAC_04572	-1.43951	9.05E-20	down	beta-lactamase
MAC_06624	-1.42874	3.67E-09	down	glycosyltransferase
MAC_00317	-1.42297	0.009226	down	glucose-methanol-choline oxidoreductase
MAC_04894	-1.41466	1.86E-11	down	flavohemoprotein
MAC_01451	-1.40273	0.026404	down	integral membrane protein
MAC_09461	-1.4012	0.033737	down	lactonohydrolase
MAC_09450	-1.39385	1.14E-16	down	sugar transporter
MAC_02546	-1.39315	3.98E-05	down	penicillin-binding protein, putative
MAC_04771	-1.38758	1.56E-06	down	DUF1275 domain protein
MAC_02366	-1.38525	7.18E-08	down	cytochrome c oxidase copper chaperone Cox17, putative
MAC_02849	-1.37944	0.027801	down	HEAT repeat protein
MAC_06932	-1.36471	4.57E-06	down	protein HMF1

MAC_09819	-1.35976	2.77E-10	down	GNS1/SUR4 family protein
MAC_01701	-1.35966	0.009286	down	putative histidine acid phosphatase
MAC_05897	-1.35658	1.42E-04	down	thiamine pyrophosphokinase
MAC_07187	-1.35609	5.07E-14	down	phosphoglucomutase 2
MAC_06330	-1.35023	0.001684	down	hypothetical protein
MAC_01774	-1.35007	0.01231	down	hypothetical protein
MAC_06275	-1.34666	0.002762	down	hypothetical protein
MAC_02098	-1.34662	5.01E-21	down	integral membrane protein
MAC_07001	-1.33961	0.049968	down	peptidyl-prolyl cis/trans isomerase
MAC_09316	-1.33345	1.41E-07	down	elastinolytic metalloproteinase Mep
MAC_02882	-1.33331	4.00E-14	down	covalently-linked cell wall protein
MAC_02232	-1.33062	1.79E-06	down	cation diffusion facilitator 1
MAC_00715	-1.32289	0.020593	down	hypothetical protein
MAC_03213	-1.31999	7.81E-15	down	hypothetical protein
MAC_03977	-1.31663	2.24E-14	down	amino acid transporter, putative
MAC_05007	-1.31514	2.46E-13	down	arrestin domain-containing protein
MAC_04142	-1.31309	4.23E-13	down	C2H2 transcription factor (AmdA), putative
MAC_06276	-1.31255	0.039906	down	hexokinase-1
MAC_04500	-1.31109	3.43E-04	down	N-acetylglucosamine-6-phosphate deacetylase
MAC_02194	-1.30574	0.00641	down	VHS domain protein
MAC_08091	-1.30279	1.08E-20	down	F-actin-capping protein subunit alpha
MAC_08796	-1.30178	1.60E-06	down	hypothetical protein
MAC_06849	-1.2945	2.39E-17	down	short-chain dehydrogenase, putative
MAC_07045	-1.2929	6.19E-13	down	hypothetical protein
MAC_02133	-1.29086	1.83E-04	down	protein disulfide-isomerase
MAC_03252	-1.29067	8.82E-06	down	hypothetical protein
MAC_09592	-1.28953	1.99E-18	down	hypothetical protein
MAC_04947	-1.28591	1.30E-08	down	phosphatidate cytidylyltransferase family protein
MAC_00603	-1.28433	1.46E-16	down	hypothetical protein
MAC_05752	-1.26717	1.41E-14	down	tyrosinase domain protein
MAC_03509	-1.26267	3.60E-14	down	UPF0145 domain protein
MAC_07817	-1.26261	5.29E-04	down	hypothetical protein
MAC_03527	-1.26244	4.60E-11	down	hypothetical protein
MAC_03782	-1.26112	1.01E-11	down	proline rich protein 5MeD
MAC_01110	-1.25991	2.12E-12	down	methylmalonate semialdehyde dehydrogenase
MAC_08962	-1.25852	1.12E-07	down	hypothetical protein
MAC_03832	-1.25793	5.97E-06	down	amidohydrolase family protein
MAC_00623	-1.2567	2.60E-15	down	hypothetical protein
MAC_03504	-1.25597	4.09E-06	down	putative WD40 domain-containing protein

MAC_04755	-1.25497	0.014241	down	secreted protein
MAC_01212	-1.24774	0.020426	down	DUF1212 domain membrane protein
MAC_04171	-1.24484	3.15E-18	down	multidrug resistant protein
MAC_03517	-1.24347	1.66E-08	down	hypothetical protein
MAC_02384	-1.23785	2.06E-13	down	hypothetical protein
MAC_05276	-1.23621	1.51E-15	down	aminotransferase, putative
MAC_09821	-1.23289	3.32E-13	down	potassium channel
MAC_08917	-1.23104	3.30E-05	down	putative flavin-dependent halogenase/O-methyltransferase
MAC_05505	-1.23026	1.10E-10	down	arsenite efflux transporter, putative
MAC_04547	-1.22964	1.98E-07	down	UPF0187 domain membrane protein
MAC_02029	-1.22845	0.001021	down	N,O-diacetyl muramidase, putative
MAC_04225	-1.2257	0.032084	down	hypothetical protein
MAC_04118	-1.21801	1.63E-12	down	C6 transcription factor, putative
MAC_01539	-1.21774	2.52E-08	down	hypothetical protein
MAC_05163	-1.21164	0.041938	down	metallo-beta-lactamase domain protein
MAC_05170	-1.21032	9.96E-13	down	acetoacetyl-CoA synthase
MAC_07568	-1.20785	0.038808	down	polyketide synthase, putative
MAC_03793	-1.20714	2.93E-18	down	2OG-Fe(II) oxygenase family oxidoreductase, putative
MAC_01686	-1.2063	1.51E-11	down	alanine racemase
MAC_05172	-1.20538	2.91E-26	down	MFS transporter, putative
MAC_03472	-1.20516	2.01E-06	down	hypothetical protein
MAC_09504	-1.20514	1.25E-05	down	F-actin capping protein beta subunit
MAC_08680	-1.20185	0.001735	down	glutamine:fructose-6-phosphate amidotransferase
MAC_02852	-1.20058	3.29E-12	down	hypothetical protein
MAC_00523	-1.20003	6.59E-10	down	hypothetical protein
MAC_02692	-1.1994	5.39E-09	down	hypothetical protein
MAC_04032	-1.19929	0.013537	down	elongation factor 3
MAC_08591	-1.19569	1.10E-04	down	sugar transporter family protein
MAC_03069	-1.19395	1.92E-04	down	hypothetical protein
MAC_05171	-1.19321	3.59E-31	down	glutathione-S-transferase theta, GST, putative
MAC_04881	-1.19289	2.37E-20	down	putative methionine permease
MAC_07567	-1.19185	0.00133	down	transporter-like protein
MAC_02099	-1.19115	4.23E-14	down	hypothetical protein
MAC_03259	-1.18948	0.007611	down	hypothetical protein
MAC_00501	-1.1884	7.82E-12	down	hypothetical protein
MAC_01827	-1.18777	2.30E-12	down	putative nuclease S1 precursor
MAC_08197	-1.1833	4.79E-11	down	hypothetical protein
MAC_07623	-1.18131	5.66E-09	down	alginate lyase
MAC_07131	-1.17736	1.18E-05	down	ser/Thr protein phosphatase superfamily

MAC_04944	-1.17699	2.93E-14	down	phosphoglycerate mutase family protein, putative
MAC_05816	-1.17312	1.34E-05	down	hypothetical protein
MAC_09529	-1.16789	0.015322	down	hypothetical protein
MAC_04772	-1.16545	3.05E-15	down	hypothetical protein
MAC_03861	-1.16096	1.77E-12	down	subtilisin-like protease
MAC_01478	-1.16048	3.01E-04	down	bZIP transcription factor
MAC_09768	-1.15546	5.42E-08	down	Quinate dehydrogenase
MAC_00216	-1.15254	3.98E-05	down	HET domain protein, putative
MAC_00957	-1.15136	0.002057	down	integral membrane protein
MAC_06209	-1.14498	3.34E-12	down	Alternative oxidase
MAC_04466	-1.14485	6.69E-23	down	hypothetical protein
MAC_04850	-1.14206	7.60E-13	down	aureobasidin-resistance protein, putative
MAC_04283	-1.13636	1.34E-08	down	glutathione-dependent formaldehyde-activating GFA
MAC_06850	-1.13609	0.001393	down	hypothetical protein
MAC_05261	-1.13592	1.75E-08	down	hypothetical protein
MAC_02531	-1.13465	1.35E-06	down	homocitrate synthase
MAC_09815	-1.13441	0.005322	down	hypothetical protein
MAC_03198	-1.13346	8.09E-16	down	GPI anchored protein, putative
MAC_06675	-1.13249	0.013678	down	C6 transcription factor, putative
MAC_06547	-1.12944	3.49E-17	down	hypothetical protein
MAC_05137	-1.12907	9.66E-07	down	hypothetical protein
MAC_06995	-1.12593	5.30E-17	down	transcription factor (Snd1/p100), putative
MAC_06259	-1.1242	1.91E-06	down	hypothetical protein
MAC_03347	-1.12116	8.59E-14	down	pirin domain protein, putative
MAC_06973	-1.121	0.030635	down	Rec8
MAC_08253	-1.12097	2.53E-11	down	6-phosphofructokinase
MAC_08915	-1.12082	0.00715	down	UBA/TS-N domain containing protein
MAC_09662	-1.12004	0.020701	down	hypothetical protein
MAC_08978	-1.11575	5.16E-11	down	hypothetical protein
MAC_08573	-1.11068	3.39E-19	down	4-aminobutyrate aminotransferase
MAC_00293	-1.11053	2.82E-16	down	calcineurin-like phosphoesterase, putative
MAC_00732	-1.10921	1.51E-14	down	mucoidy inhibitor-like protein
MAC_03335	-1.1006	2.23E-08	down	hypothetical protein
MAC_09006	-1.09625	9.74E-05	down	allantoate permease
MAC_00391	-1.0959	8.60E-17	down	neutral alpha-glucosidase ab precursor
MAC_05847	-1.09398	1.40E-04	down	hypothetical protein
MAC_00891	-1.09344	0.007983	down	hypothetical protein
MAC_05507	-1.09304	4.89E-09	down	arsenate reductase (Arc2), putative
MAC_04428	-1.09012	4.21E-26	down	acetyltransferase, GNAT family

MAC_05383	-1.0897	1.77E-08	down	phosphate permease
MAC_05773	-1.0885	1.41E-06	down	K(+)/H(+) antiporter 1
MAC_03667	-1.08809	0.001664	down	alpha-1,2-mannosidase, putative subfamily
MAC_07019	-1.08737	8.99E-12	down	hypothetical protein
MAC_01012	-1.08471	0.01778	down	dipeptidyl peptidase
MAC_04336	-1.08446	0.011897	down	glycosyl hydrolase
MAC_06775	-1.08389	2.40E-06	down	glutathione-dependent formaldehyde-activating
MAC_09362	-1.08372	1.26E-13	down	cytochrome P450 78A3
MAC_02541	-1.08114	7.02E-13	down	hypothetical protein
MAC_02039	-1.08037	2.04E-17	down	quinone oxidoreductase
MAC_00960	-1.07912	1.53E-07	down	COP9 signalosome complex subunit 5
MAC_08912	-1.0785	6.23E-12	down	cysteine-rich secreted protein
MAC_04282	-1.07589	2.53E-25	down	epoxide hydrolase 1
MAC_00185	-1.07435	2.26E-08	down	beta-mannosidase
MAC_01848	-1.074	0.012562	down	D-isomer specific 2-hydroxyacid dehydrogenase
MAC_08284	-1.07398	5.62E-29	down	ferric-chelate reductase (Fre2), putative
MAC_06304	-1.07011	2.36E-14	down	thioesterase family protein
MAC_04272	-1.06749	0.049085	down	hypothetical protein
MAC_07718	-1.06443	2.25E-06	down	phthalate transporter
MAC_01006	-1.06434	3.68E-14	down	putative pirin
MAC_09637	-1.06246	1.37E-09	down	hypothetical protein
MAC_07174	-1.06195	1.82E-08	down	feruloyl esterase B precursor
MAC_07943	-1.061	6.78E-12	down	hypothetical protein
MAC_05025	-1.05872	3.55E-12	down	hypothetical protein
MAC_05426	-1.05646	2.03E-12	down	hypothetical protein
MAC_04110	-1.05511	1.13E-16	down	hypothetical protein
MAC_09510	-1.05497	1.32E-14	down	integral membrane protein, putative
MAC_01127	-1.05401	2.34E-10	down	putative 3-oxoadipate enol-lactonase I
MAC_04274	-1.05244	4.89E-06	down	histidine acid phosphatase, putative
MAC_00235	-1.05001	7.01E-13	down	3-hydroxyacyl-CoA dehydrogenase, putative
MAC_01566	-1.04976	1.77E-14	down	hypothetical protein
MAC_01735	-1.04761	5.26E-09	down	hypothetical protein
MAC_09778	-1.04306	0.017828	down	benzoate 4-monooxygenase cytochrome P450
MAC_07331	-1.0416	0.012185	down	V8-like Glu-specific endopeptidase
MAC_01846	-1.04039	1.22E-09	down	heterokaryon incompatibility protein
MAC_04855	-1.0401	9.14E-04	down	oxidoreductase
MAC_06774	-1.03769	1.84E-10	down	hypothetical protein

MAC_05449	-1.03677	6.17E-21	down	aminoadipate-semialdehyde dehydrogenase, putative
MAC_00477	-1.03643	1.17E-09	down	facilitated glucose transporter, putative
MAC_05147	-1.03575	0.007558	down	plasma membrane calcium-transporting ATPase 2
MAC_01283	-1.03475	4.45E-08	down	ATP-dependent RNA helicase DED1
MAC_01989	-1.03397	2.72E-07	down	hypothetical protein
MAC_08673	-1.03215	8.75E-04	down	peptidoglycan binding domain containing protein
MAC_01278	-1.03145	0.02021	down	phenylalanine ammonia-lyase, putative
MAC_00567	-1.02895	6.79E-10	down	hypothetical protein
MAC_06773	-1.02731	7.38E-11	down	golgi reassembly-stacking protein 2
MAC_04455	-1.02646	0.019829	down	succinate semialdehyde dehydrogenase
MAC_02647	-1.02519	0.003236	down	C6 transcription factor, putative
MAC_07642	-1.02446	2.00E-08	down	ubiE/COQ5 methyltransferase, putative
MAC_06588	-1.02435	2.49E-09	down	acyltransferase, putativ
MAC_08195	-1.02434	8.38E-09	down	initiation-specific alpha-1,6-mannosyltransferase
MAC_09613	-1.02403	3.44E-16	down	type I phosphodiesterase/nucleotide pyrophosphatase
MAC_01909	-1.02314	2.18E-06	down	NlpC/P60-like cell-wall peptidase
MAC_08976	-1.02311	2.12E-04	down	hypothetical protein
MAC_05547	-1.02211	3.70E-08	down	proteinase, putative
MAC_03665	-1.02113	2.16E-06	down	hypothetical protein
MAC_06169	-1.01827	3.44E-07	down	arginine transporter
MAC_07974	-1.01335	4.26E-13	down	hypothetical protein
MAC_05950	-1.0113	2.66E-04	down	hypothetical protein
MAC_05310	-1.01091	2.49E-13	down	acetyl-coenzyme A synthetase
MAC_02310	-1.00988	2.57E-10	down	disulfide isomerase
MAC_08583	-1.00952	9.67E-15	down	hypothetical protein
MAC_02658	-1.00814	0.037881	down	hypothetical protein
MAC_07035	-1.00764	2.69E-04	down	cytochrome P450 phenylacetate 2-hydroxylase, putative
MAC_06576	-1.00736	1.32E-10	down	DUF636 domain protein
MAC_08424	-1.00615	1.01E-10	down	MFS transporter (Mch2), putative
MAC_00958	-1.00357	0.028087	down	hypothetical protein
MAC_06577	-1.00309	1.49E-06	down	carbohydrate-binding protein
MAC_09284	-1.00295	2.60E-07	down	fungal specific transcription factor, putative
MAC_04023	-1.00279	0.010906	down	sphinganine hydroxylase Sur2, putative
MAC_01437	-1.00184	1.38E-08	down	putative O-methyltransferase
MAC_02470	1.003316	1.17E-10	up	MAK16 protein, putative

MAC_09398	1.006204	1.49E-05	up	integral membrane protein
MAC_02812	1.014361	6.42E-11	up	vacuolar protein-sorting protein BRO1
MAC_04745	1.01536	2.21E-11	up	phospholipase PldA, putative
MAC_03607	1.01813	1.27E-15	up	putative pig glycolipid transfer protein
MAC_08844	1.01844	2.16E-08	up	salicylate hydroxylase
MAC_06853	1.019146	3.12E-13	up	NADPH-P450 reductase
MAC_02059	1.029153	4.05E-09	up	putative cholesterol oxidase precursor
MAC_07911	1.031693	3.02E-04	up	haloalkanoic acid dehalogenase
MAC_06726	1.031876	1.08E-09	up	trypsin-related protease
MAC_08774	1.036943	4.95E-04	up	hypothetical protein
MAC_08720	1.043944	0.014211	up	cell wall protein
MAC_04688	1.045505	2.36E-08	up	flavohemoglobin
MAC_06381	1.04737	8.81E-06	up	Lcc1; ascorbase & Cu-oxidase
MAC_01193	1.05003	0.002023	up	acyl-CoA oxidase, putative
MAC_09033	1.05019	1.85E-09	up	hypothetical protein
MAC_03210	1.051143	4.16E-04	up	geranylgeranyl diphosphate synthase
MAC_01338	1.053023	7.55E-22	up	P450 monooxygenase
MAC_08470	1.054462	0.010305	up	P450 monooxygenase
MAC_07387	1.055541	1.53E-10	up	hypothetical protein
MAC_02378	1.059105	1.26E-07	up	hypothetical protein
MAC_09272	1.059723	4.15E-08	up	peptide synthetase 3
MAC_04898	1.060227	1.76E-19	up	putative oxidoreductase
MAC_05160	1.061332	4.24E-21	up	peptide synthetase
MAC_03690	1.062719	1.94E-14	up	putative elymoclavine monooxygenase
MAC_04810	1.06342	5.27E-09	up	putative catalase
MAC_07240	1.063427	1.40E-07	up	oxidoreductase
MAC_05987	1.067888	5.87E-12	up	oxidoreductase
MAC_04763	1.073906	2.36E-12	up	ergot alkaloid biosynthetic protein B
MAC_07319	1.076621	5.84E-17	up	putative ergot alkaloid biosynthetic protein A
MAC_01182	1.077211	4.74E-07	up	putative dimethyl-allyl-tryptophan-synthase
MAC_00787	1.079339	2.22E-29	up	monooxygenase
MAC_00764	1.08089	0.005431	up	putative glutathione S-transferase
MAC_01598	1.081355	1.23E-12	up	DUF614 domain protein
MAC_04433	1.082694	5.11E-12	up	hypothetical protein
MAC_03855	1.083942	8.96E-19	up	kinase-related protein
MAC_03034	1.092582	1.99E-15	up	glycerol kinase
MAC_06691	1.096273	9.17E-17	up	phosphate transporter
MAC_04388	1.096663	4.60E-04	up	hypothetical protein
MAC_08845	1.098078	1.33E-07	up	hypothetical protein
MAC_08904	1.105145	2.35E-15	up	phosphatidyl inositol-specific phospholipase C
MAC_00066	1.111479	1.34E-04	up	esterase, putative

MAC_09233	1.111549	6.10E-11	up	hypothetical protein
MAC_03496	1.114136	1.45E-09	up	monooxygenase
MAC_09649	1.115641	0.012927	up	inositolphosphorylceramide-B C-26 hydroxylase
MAC_06299	1.122143	0.00463	up	cystein rich protein
MAC_00973	1.122209	0.01064	up	putative cell wall glycoprotein
MAC_02841	1.124152	0.002329	up	PQQ repeat containing protein
MAC_01760	1.1249	5.59E-07	up	cholinesterase
MAC_00595	1.125398	8.47E-30	up	amidohydrolase family protein
MAC_02556	1.126404	0.007858	up	polysaccharide lyase family 8 protein
MAC_00734	1.127557	4.44E-11	up	hypothetical protein
MAC_09268	1.127783	5.46E-14	up	4-coumarate-CoA ligase 2
MAC_04109	1.129577	1.70E-10	up	hypothetical protein
MAC_04517	1.133032	3.09E-11	up	ankyrin repeat protein
MAC_08411	1.136625	3.40E-08	up	hypothetical protein
MAC_07424	1.137978	7.92E-15	up	Prolyl endopeptidase
MAC_03099	1.139193	1.00E-11	up	hypothetical protein
MAC_02251	1.147773	0.008308	up	salicylate hydroxylase, putative
MAC_00860	1.157046	4.96E-04	up	hypothetical protein
MAC_04234	1.157524	0.002978	up	hypothetical protein
MAC_08788	1.161719	7.34E-06	up	sulfatase
MAC_04815	1.161887	0.035356	up	Sugar transporter family protein
MAC_06002	1.163201	0.025896	up	Aldose 1-epimerase family protein
MAC_07325	1.16612	0.005425	up	NmrA family transcriptional regulator
MAC_03947	1.167205	2.79E-12	up	C2H2 finger domain protein, putative
MAC_04279	1.167248	6.87E-31	up	hypothetical protein
MAC_04620	1.168629	6.12E-07	up	hypothetical protein
MAC_04533	1.168993	0.002166	up	Phosphodiesterase/alkaline phosphatase D
MAC_00544	1.171803	3.22E-04	up	DUF1212 domain membrane protein Prm10
MAC_03412	1.172841	1.01E-11	up	muramidase, putative
MAC_03664	1.175326	1.41E-08	up	UPF0145 domain-containing protein
MAC_08939	1.176179	6.90E-12	up	DUF300 domain protein, putative
MAC_01183	1.179183	2.11E-29	up	cytochrome P450
MAC_06816	1.187519	1.82E-08	up	hypothetical protein
MAC_03500	1.190154	6.69E-09	up	Transmembrane amino acid transporter family protein
MAC_06728	1.195297	1.46E-25	up	NRPS-like enzyme, putative
MAC_09531	1.199206	5.38E-09	up	phosphoglycerate mutase family protein
MAC_07810	1.200656	1.32E-10	up	C6 transcription factor, putative
MAC_09263	1.205993	4.23E-18	up	hypothetical protein

MAC_03023	1.206715	0.0055	up	ATP-binding cassette transporter ABC1
MAC_05109	1.207124	2.28E-04	up	hypothetical protein
MAC_06640	1.214285	2.09E-07	up	homoserine dehydrogenase
MAC_03461	1.216893	2.55E-17	up	zinc-binding oxidoreductase ToxD, putative
MAC_04249	1.218547	2.31E-13	up	hypothetical protein
MAC_05731	1.229189	4.11E-14	up	MFS monocarboxylate transporter, putative
MAC_07745	1.236502	1.28E-09	up	pyoverdine/dityrosine biosynthesis protein, putative
MAC_08541	1.238412	5.07E-16	up	aldehyde dehydrogenase
MAC_01655	1.240169	6.67E-20	up	tryptophanyl-tRNA synthetase
MAC_08294	1.240204	3.29E-14	up	hypothetical protein
MAC_07945	1.242902	2.47E-12	up	chitooligosaccharide oxidase
MAC_07835	1.245519	2.45E-24	up	short chain dehydrogenase/reductase SDR
MAC_04231	1.248455	2.34E-19	up	hypothetical protein
MAC_02799	1.257555	2.39E-17	up	hypothetical protein
MAC_01770	1.265497	0.001862	up	siderophore iron transporter
MAC_09715	1.266338	7.43E-18	up	putative endochitinase CHI2
MAC_07971	1.270314	6.41E-27	up	hypothetical protein
MAC_04621	1.272502	1.70E-15	up	hypothetical protein
MAC_00136	1.273054	0.005199	up	hypothetical protein
MAC_06833	1.277855	6.78E-12	up	charged multivesicular body protein 3
MAC_01556	1.285902	1.65E-06	up	HET domain protein
MAC_07383	1.28649	8.24E-12	up	copper amine oxidase 1
MAC_03606	1.29131	4.42E-15	up	hypothetical protein
MAC_07845	1.306259	0.035199	up	hypothetical protein
MAC_01290	1.307843	3.66E-04	up	C2H2 finger domain protein, putative
MAC_00065	1.313586	1.33E-06	up	nitroreductase family protein
MAC_01832	1.314997	2.43E-11	up	p21 activated kinase-like protein
MAC_04599	1.31618	4.21E-20	up	hypothetical protein
MAC_03676	1.321285	1.21E-23	up	ThiJ/PfpI family protein
MAC_07378	1.32196	8.60E-11	up	FAD binding domain protein
MAC_07251	1.325114	2.18E-18	up	NUDIX domain-containing protein
MAC_09161	1.340007	1.43E-04	up	SpoC1-C1C
MAC_05729	1.342037	4.56E-14	up	hypothetical protein
MAC_06315	1.346399	1.30E-23	up	hypothetical protein
MAC_03945	1.356347	9.80E-05	up	hypothetical protein
MAC_03378	1.358106	7.16E-25	up	glycylpeptide N-tetradecanoyltransferase
MAC_05632	1.361377	4.71E-05	up	hypothetical protein
MAC_09191	1.365899	0.006469	up	exo-1,3-beta-D-glucanase

MAC_04122	1.373253	9.36E-20	up	hypothetical protein
MAC_09122	1.379922	2.46E-14	up	hypothetical protein
MAC_03809	1.380738	1.33E-15	up	hypothetical protein
MAC_09355	1.3905	1.51E-08	up	amino acid adenylation domain protein
MAC_04137	1.419429	3.21E-08	up	hypothetical protein
MAC_02947	1.423231	8.27E-25	up	hypothetical protein
MAC_00193	1.427993	7.22E-09	up	aspartic protease precursor
MAC_06123	1.442775	2.69E-15	up	glutathione S-transferase family protein, putative
MAC_01833	1.474354	2.35E-22	up	spherulin 4-like cell surface protein
MAC_09267	1.486202	1.17E-04	up	UDP-glucose 4-epimerase, putative
MAC_01331	1.498802	3.12E-08	up	Mitochondrial import inner membrane translocase subunit tim-22
MAC_05951	1.512198	5.29E-06	up	hypothetical protein
MAC_09367	1.513705	0.008179	up	hypothetical protein
MAC_03346	1.52109	0.042125	up	cation transporter, putative
MAC_01476	1.53765	0.04386	up	phenylacetaldoxime dehydratase family protein, putative
MAC_06890	1.540153	6.41E-27	up	pyruvate decarboxylase
MAC_01716	1.547091	1.32E-20	up	protein phosphatase regulatory subunit Gac1
MAC_05199	1.550722	2.91E-05	up	26S proteasome regulatory subunit-like protein
MAC_03368	1.568931	3.07E-06	up	acyl-CoA dehydrogenase
MAC_02945	1.576092	2.22E-18	up	hexose transporter-like protein
MAC_01577	1.586497	8.24E-12	up	capsule polysaccharide biosynthesis protein
MAC_06706	1.597363	2.34E-10	up	salicylate hydroxylase
MAC_06779	1.61465	2.05E-04	up	hypothetical protein
MAC_08745	1.635359	1.05E-06	up	delta 8-(E)-sphingolipid desaturase, putative
MAC_05316	1.647052	1.98E-29	up	hypothetical protein
MAC_00325	1.654553	2.47E-04	up	alpha-1,2-mannosidase family protein, putative
MAC_00167	1.686546	0.049498	up	hypothetical protein
MAC_07133	1.686696	1.11E-23	up	aldo-keto reductase (AKR13), putative
MAC_03963	1.707676	5.85E-06	up	glyoxylate reductase
MAC_03364	1.714692	0.002632	up	BAR domain containing protein
MAC_02946	1.737124	4.77E-50	up	phosphate-repressible phosphate permease
MAC_07851	1.746981	7.79E-04	up	hypothetical protein
MAC_02294	1.764796	0.031101	up	hypothetical protein
MAC_07853	1.769912	0.002562	up	putative lipoxygenase 1
MAC_06944	1.795111	1.33E-32	up	DUF124 domain protein

MAC_05606	1.860892	7.76E-37	up	kelch repeat protein
MAC_08212	1.872731	3.38E-04	up	putative mosquitocidal toxin
MAC_08776	1.899635	2.45E-49	up	putative glucoamylase GMY2
MAC_04054	1.905558	1.61E-07	up	hydrophobin
MAC_04033	1.917345	9.34E-10	up	clock-controlled pheromone ccg-4 precursor
MAC_06941	1.920848	0.001944	up	1-phosphatidylinositol-4,5-bisphosphate phosphodiesterase gamma 2
MAC_07956	1.989893	1.94E-14	up	oxidoreductase, zinc-binding protein
MAC_08614	2.027012	0.009688	up	glucan 1,3-beta-glucosidase GLUC78 precursor
MAC_09416	2.039989	6.84E-06	up	choline transport protein
MAC_03331	2.044171	1.19E-38	up	hypothetical protein
MAC_08744	2.096391	0.048057	up	SH3 domain protein
MAC_09034	2.126875	4.72E-43	up	hypothetical protein
MAC_00228	2.158279	1.61E-04	up	aldo-keto reductase (AKR), putative
MAC_06925	2.163495	0.023714	up	nucleoside-diphosphate-sugar epimerase, putative
MAC_02290	2.171815	0.040085	up	hypothetical protein
MAC_01194	2.195933	1.27E-05	up	myosin-crossreactive antigen
MAC_07294	2.253955	1.20E-21	up	hypothetical protein
MAC_08230	2.269763	1.08E-04	up	subtilisin-like protease
MAC_07813	2.287238	7.90E-23	up	Amino-acid permease inda1
MAC_06945	2.588378	1.46E-23	up	hypothetical protein
MAC_00801	2.897788	2.45E-14	up	hypothetical protein
MAC_02438	2.955837	0.011198	up	fatty acid-binding protein FABP, putative
MAC_03018	2.964929	5.76E-14	up	putative D-galacturonic acid reductase
MAC_04050	4.052927	2.10E-88	up	HAD superfamily hydrolase, putative

Table S3 DEGs of Δ*MaAts* vs. WT involved in stress tolerances and cell wall components

Gene ID	Product name	Function description	Log_2^{ratio} (Δ*MaAts*/WT)	References
MAC_00623	β-glucosidase	Related to thermostability	-1.25669815	[1]
MAC_07727	C2H2 finger domain protein, putative	Strongly up-regulated by cold, drought and heat stresses	-2.9225414	[2]
MAC_07325	PQQ repeat containing protein	PQQ inhibits melanin production	1.16611988	[3]
MAC_05632	feruloyl esterase B precursor	Involved in the rigidity and strength of the plant cell wall	1.36137658	[4]
MAC_04881	pirin domain protein, putative	Regulates secondary wall biosynthesis in *Arabidopsis*	-1.19288629	[5]
MAC_04850	transcription factor (Snd1/p100), putative	Involved in the regulation of secondary wall biosynthesis in *Arabidopsis*	-1.1420566	[5]
MAC_00235	phosphoinositide 3-phosphate phosphatase	Contributes to the wall thickness of fiber cells and vessel elements in yeast	-1.05000664	[6]
MAC_06774	phospholipase PldA, putative	Related to cell wall integrity	-1.0376872	[7]
MAC_00567	putative maltase MLT2	Related to in stress tolerance and cell wall integrity	-1.0289541	[8]
MAC_01213	oxidoreductase, 2OG-Fe(II) oxygenase family, putative	Relate to cell wall formation	-1.6598324	[9]
MAC_01513	RTA1 domain protein, putative	Relate to cell wall	-2.01017551	[10]
MAC_02366	glycerophosphoryl diester phosphodiesterase family protein	Related to the rigidity of the cell wall	-1.3852548	[11]
MAC_02951	sugar transporter	Maintaining cell wall integrity	-2.2093985	[12]
MAC_06773	vacuolar protein-sorting protein BRO1	Involved in the regulation of secondary wall biosynthesis in *Arabidopsis*	-1.02731249	[13]

References

1. Kengen, S.W.; Luesink, E.J.; Stams, A.J.; Zehnder, A.J. Purification and characterization of an extremely thermostable *β-glucosidase* from the hyperthermophilic archaeon *Pyrococcus furiosus*. *Eur. J. Biochem*. **2010**, *213*, 305–312.

2. Zhang, X.; Zhang, B.; Li, M.J.; Yin, X.M.; Huang, L.F.; Cui, Y.C.; Wang, M.L.; Xia, X.J. *OsMSR15* encoding a rice C2H2-type zinc finger protein confers enhanced drought tolerance in transgenic Arabidopsis. *J. Plant. Biol*. **2016**, *59*, 271–281.

3. Sato, K.; Toriyama, M. Effect of pyrroloquinoline quinone (PQQ) on melanogenic protein expression in murine B16 melanoma. *J. Dermatol. Sci.* **2009**, *53*, 140–145.

4. Mathew, S.; Abraham, T.E. Ferulic acid: an antioxidant found naturally in plant cell walls and feruloyl esterases involved in its release and their applications. *Crit. Rev. Biotechnol*. **2004**, *24*, 59–83.

5. Zhong, R.; Ye, R.Z.H. The MYB46 transcription factor is a direct target of SND1 and regulates secondary wall biosynthesis in *Arabidopsis*. *Plant Cell* **2007**, *19*, 2776–2792.
6. Zhong, R. Mutation of SAC1, an Arabidopsis SAC Domain Phosphoinositide Phosphatase, Causes Alterations in Cell Morphogenesis, Cell Wall Synthesis, and Actin Organization. *Plant Cell* **2005**, *17*, 1449–1466.
7. May, K.L.; Silhavy, T.J.; Gottesman, S. The *Escherichia coli* phospholipase PldA regulates outer membrane homeostasis via lipid signaling. *mBio*. **2018**, *9*, e00379-18.
8. Wang, X.; Yang, Z.; Wang, M.; Meng, L.; Jiang, Y.; Han, Y. The *BRANCHING ENZYME1* gene, encoding a glycoside hydrolase family 13 protein, is required for in vitro plant regeneration in *Arabidopsis*. *Plant Cell Tiss. Org.* **2014**, *117*, 279–291.
9. Fang, L.; Zhao, F.; Cong, Y.; Sang, X.; Du, Q.; Wang, D.; Li, Y.; Ling, Y.; Yang, Z.; He, G. Rolling-leaf14 is a 2OG-Fe (II) oxygenase family protein that modulates rice leaf rolling by affecting secondary cell wall formation in leaves. *Plant Biotechnol. J.* **2012**, *10*, 524–532.
10. Johnson, S.S.; Hanson, P.K.; Manoharlal, R.; Brice, S.E.; Cowart, L.A.; Moye-Rowley, W.S. Regulation of yeast nutrient permease endocytosis by ATP-binding cassette transporters and a seven-transmembrane protein, RSB1. *J. Biol. Chem*. **2010**, *85*, 35792–35802.
11. Hayashi, S. The glycerophosphoryl diester phosphodiesterase-like proteins SHV3 and its homologs play important roles in cell wall organization. *Plant Cell Physiol*. **2008**,*49*, 1522–1535.
12. Song, X.; Zhang, B.; Zhou, Y. Golgi-localized UDP-glucose transporter is required for cell wall integrity in rice. *Plant Signal. Behav*. **2011**, *6*, 1097–1100.
13. Nickas, M.E.; Yaffe, M.P. *BRO1*, a novel gene that interacts with components of the Pkc1p-mitogen-activated protein kinase pathway in *Saccharomyces cerevisiae*. *Mol. Cell. Biol*. **1996**, *16*, 2585–2593.
